# High resolution 2D beam steerer made from cascaded 1D liquid crystal phase gratings

**DOI:** 10.1038/s41598-022-09201-0

**Published:** 2022-03-24

**Authors:** Mario García de Blas, Javier Pereiro García, Sergio Vera Andreu, Xabier Quintana Arregui, Manuel Caño-García, Morten Andreas Geday

**Affiliations:** CEMDATIC, ETSI Telecomunicación, Universidad Politécnica de Madrid, Av. Complutense 30, 28040 Madrid, Spain

**Keywords:** Liquid crystals, Photonic devices, Applied optics, Optical physics

## Abstract

Optical beam steering (BS) has multiple applications in fields like target seeking and tracking, optical tweezers, billboard displays and many others. In this work, a two-dimensional beam deflector based on blaze gratings is presented. Phase-only 1D blaze gratings have been prepared using maskless Direct Laser Writing (DLW) resulting in high-resolution structures in indium-tin oxide (ITO) coated glass wafers. The device is composed of two identical 1D liquid crystal (LC) cells cascaded orthogonally back-to-back, with a resultant active area of 1.1 × 1.1 mm^2^. The 1D cells have been prepared with 144 pixels each with a 7.5 µm pitch. The total 288 pixels are driven by a custom made 12-bit Pulse Width Modulation (PWM) electronic driver, allowing for an arbitrarily high resolution. The system performance is documented, and the efficiency of the system has been tested. A maximum diagonal steering angle of ± 3.42° was achieved.

## Introduction

Redirecting an incoming light beam to a desired direction is used in a plethora of applications where this technology is relevant such as optical tweezers^1^, optical communications^2–5^, light ranging ^6–8^ and augmented reality^9^.

Beam steerers (BSs) can be divided into two categories, depending on whether the deviation is done by a mechanical or non-mechanical control. Some mechanical approaches make use of micro-mirror or micro-lenses moved by using elements such as piezo actuators^10,11^ or micro-electromechanical system^12–14^.

In the case of space communications, a non-mechanical system might be beneficial as no inertia is used to redirect the communication wave link, and no counterbalancing is needed. Non-mechanical methods include: electro-optics and acoustic-optics deflectors^15^ and spatial light modulators (optical phase arrays that allow for addressing individual pixels)^16,17^.

Liquid Crystals (LCs) for non-mechanical beam deflectors exploit the electrooptical properties of this material, as it can quickly reorientate its molecules when an external electric field is applied^18,19^. The simplest*, positive nematic*, LC is a birefringent material characterized by two refractive indices (ordinary and extraordinary) and a dielectric anisotropy with the same symmetry. When an electric field is applied to the LC the molecules will tend to align with the field vectors, resulting in an effective reorientation—*switching*—of the optical parameters. In LC devices the switching plane is defined by a preferred alignment direction, obtained by one of various techniques^20,21^ and the applied field. Controlling the external field, it is possible to arbitrarily change the effective extraordinary index by switching the LC partially, as discussed in literature^22^. Having a finite thickness means that light polarized along switching plane will experience a phase retardation depending on the switching state.

LC are widely used for optical adaptative elements others than BS, for example flat tunable lenses^23–25^. LC beam steerers can be considered as phase-only devices as they interact with light modifying their phase leaving other characteristics changeless^22^. The objective of many studies about LC beam steerers is to improve the response-time of the device, increase the deviation angle and improve the efficiency of the process^26^. Many different LC configurations for beam steering devices have been described such as Polarization Gratings (PG)^27–29^, Pancharatnam–Berry phase devices^30^, Optical Phase Arrays (OPA)^31–33^ and Liquid Crystal On Silicon (LCoS)^34,35^. Many different LC configurations for beam steering devices have been described such as Polarization Gratings^28,29^, Pancharatnam-Berry phase devices^30^, Optical Phase Arrays (OPAs)^31–33^ and Liquid Crystal On Silicon (LCoS)^34,35^ as reviewed in He et al. (2019)^18^. In this review a detailed comparison of high performance OPAs^36^, PGs^37,38^, Volume Bragg gratings^39^ is performed.

Those deflectors in which the active area includes electronics, such as the LCoS and active-matrix thin-film transistor, cannot be adequately shielded for ionizing radiation environments. Making them unsuitable for space applications. Passive matrix LC transmissive systems have been tested under ionization radiation and their inertness has been demonstrated^40^.

An LC BS can be divided into analog or digital devices. An analog LC device is similar to a refractive prism, as a continuous refractive index gradient in a LC cell if adequately designed. Analog LC devices have the advantage of continuous angular variation of the incoming ray, although they do not achieve wide maximum angles. On the other hand, digital beam steerers, often diffraction phase gratings, have the drawback of switching between fixed points, not achieving a continuous deviation of the incoming ray. A combination of both, refractive and diffractive components, can lead to a fine tunable device^41^.

The present work combines a series of scientific-technical progress gained in previous publications. It employs both the resolution and the fine tuning presented by Oton et al.^41^, while at the same time reducing the manufacturing complexity using laser ablation rather than conventional^40^ or multi-layer^41^ photolithography without compromising the fill factor of the cell. Furthermore, combining the new device with the driver presented in García de Blas et al.^42^ means that it can be operated in voltage ranges where the LC retardation is not linearly dependent on the applied voltage when generating blaze gratings with fractional period of electrodes. With respect to the most recent paper^42^, mayor improvements are presented; the number of electrodes, lithographic resolution and angular range of the device have been doubled. Furthermore, and most importantly, the new 2D device is made of two *identical* LC cells with a switching plane, at an angle of 45° to the linear pixels, which improves drastically the manufacturing yield.

Each electrode in the active area is individually addressed, unlike larger, high-resolution devices presented in previous works by others^43^, leading to a BS with an arbitrarily small resolution. The arbitrary high angular resolution is demonstrated as examples of images mapped as points projected onto a screen.

## Diffraction theory

**Figure 1:**
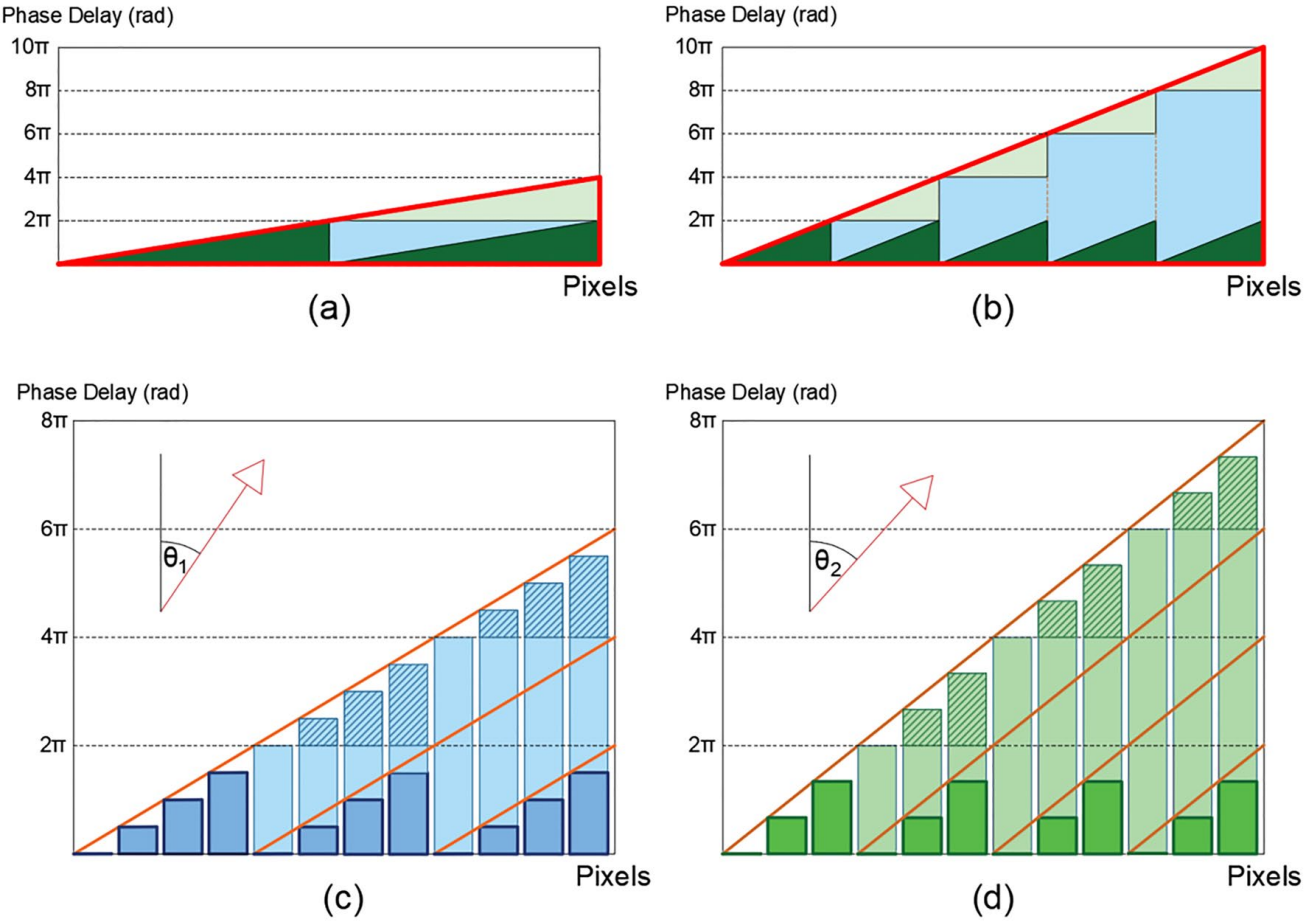
Schematics of a 2π phase wrapping and the discretization of continuous phase retardation. (**a**) The red triangle represents a prism with a maximum phase delay of 4π. Applying a 2π phase wrapping results in a periodic grating of two identical prisms (green triangles) comparable, in phase delay, with the original one. (**b**) The red triangle represents a prism with a maximum phase delay of 10π. Applying a 2π phase wrapping results in a periodic grating of 5 identical prisms (green triangles). (**c**) Schematic of the discretization of a continuous retardation of 6π, over 12 pixels, applying a 2π phase wrapping. The result is a periodic phase grating structure with period equal to 3 (k = 4). (**d**) Schematic of the discretization of a continuous retardation of 8π, over 12 pixels, applying a 2π phase wrapping. The result is a periodic phase grating structure with period equal to 4 (k = 3). As the slope of the grating is increased (orange lines), the deviation angle is increased ${(\theta }_{2}> {\theta }_{1})$.

Applying Fresnel's theory one can construct a periodic diffraction grating that deflects a beam of light analogously to how a prism does. By dividing the prism into periodic sections with a phase variation of up to 2π and eliminating the phase delays that constitute multiple delays of 2π (phase wrapping), a periodic diffraction blaze grating that deflects light an angle equal to that of the original prism is produced (Fig. 1a). To make a diffraction grating that deviates a greater angle, the width of the grating period must be decreased and consequently the slope of the grating prisms will increase (Fig. 1b). Therefore, the variation of the angle of deviation of light (to follow the slope of the original prism) requires an arbitrary variation of the period.

To manufacture a tunable periodic diffraction grating with LC, a discretization must be performed, since the size of the pixels cannot be arbitrarily small (they typically have a width of several times the wavelength). This introduces a constrain on the maximum angle of deviation, which is achieved with a binary grating, with phase delay pattern of 0, π, 0, π,… Discretization and the effect of the interpixel (called fringing effect) introduce a loss of efficiency in beam deflection.

The steering angle produced by such a blazed grating is described in Eq. 1^44^:

1 $$\theta =arcsin\left(\frac{\lambda }{\Delta }\right)=arcsin\left(\frac{\lambda }{k*pixel pitch}\right)$$

where λ is the wavelength of the incoming wave and Δ is equal to the total number of pixels per period (k) times the *pixel pitch*.

Discrete deviation angles can be achieved, employing periods of 2, 3, 4,…,*n* pixels (Fig. 1c,d). With decreasing the angle, *i.e.* increasing number of pixels (*k*) in each period, the separation between deviated angles for successive values of *k* is reduced, and the discrete approximation to the continuous prism is improved. Hence, for high numbers of *k* a high efficiency quasi-continuous beam steering tuning can be achieved. For small values of *k* (wide angle steering) the approximation to the ideal prism is compromised, and the angular separation between the steering angle for successive values of *k* increases.

**Figure 2:**
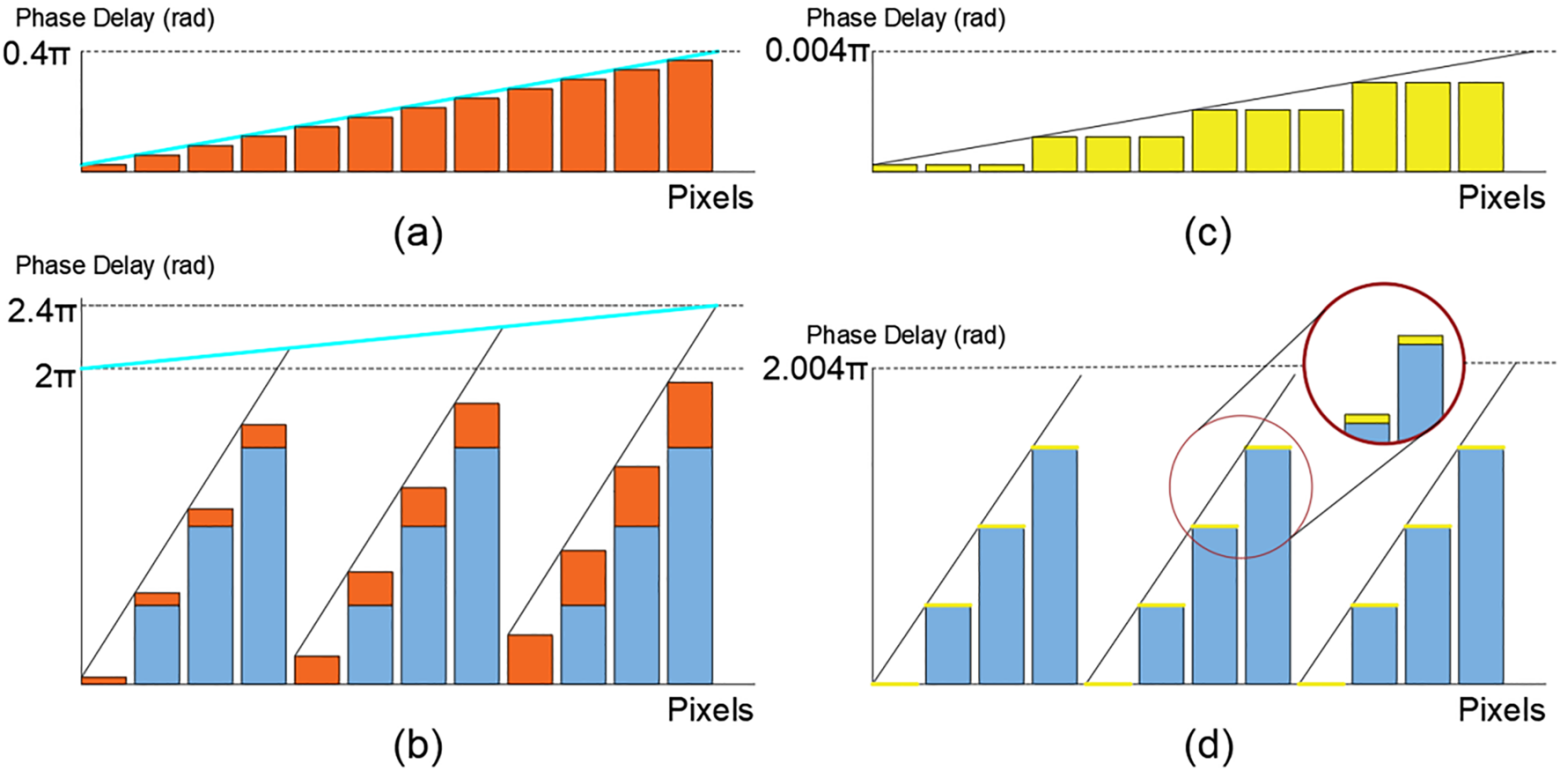
Schematic of the combination of two gratings to generate non-periodic grating structures. (**a**) Schematic of the discretization of a continuous retardation of a 0.4π delay, over 12 pixels. In this case, no phase wrapping is needed as the maximum phase delay is less than 2π. (**b**) Schematic of the combination of a continuous phase delay of 0.4π (**a**) and a wrapped grating structure of period equal to 3 (Fig. 1c). The result is a non-periodic phase grating structure. (**c**) Schematic of a discretized diffraction grating of a continuous retardation of 0.004π delay, over 12 pixels. In this case, there is a binning of three pixels in every phase step. No phase wrapping is needed as the maximum phase delay is less than 2π. (**d**) Schematic of the combination of a continuous phase delay of 0.004π (**c**) and a wrapped grating structure of period equal to 3 (Fig. 1c). The result is a non-periodic phase grating structure.

However, a tunable LC diffraction grating with quasi-continuous beam steering over the entire range of deflection can be achieved, which is intuitively understood with an example. Consider the stacking of two diffraction gratings with the same pixel pitch. Any angle of deviation less than the discrete angle step can be achieved by adding the phase delay of successive gratings of the stacked devices. By adding the delay introduced by each of them and subtracting the integer multiples of 2π when the delay exceeds this value (phase wrapping), a delay pattern is obtained that can be addressed by a single device. The result is a non-periodic diffraction grating (Fig. 2).

In general, applying this phase wrapping procedure to a prism, any deviation angle with an arbitrary granularity in deviation angle can be achieved, only limited by the resolution of the electronics driving the individual pixels. The electronic resolution limitation is furthermore reduced by binning of pixels (Fig. 2c), albeit at a cost in diffraction efficiency.

The periods may be positive or negative, corresponding to the sign of the steering angle. The diffraction efficiency will be affected by the fringe field and crosstalk^45,46^ and by the fill factor, *i.e.* the relationship between the pixel and the inter-pixel gap^47^.

## Methods

### Manufacturing

There are many manufacturing ways to manufacture LC beam steering devices, depending on the requirements for the specific application^48,49^. In the case of passive blazed gratings they can be manufactured , for instance, using a deposition and a photolithography process^50,51^. Here the two-dimensional BS is made up of two identical cascaded 1D cells. Each cell has two indium-tin oxide (ITO) coated glass substrates in a sandwich-like configuration. One of them is ablated with a Direct Laser Writing (DLW) technique, using a back-scribing approach, to transfer a pixelated pattern onto the ITO surface^52^. An UV 300 mW, 349 nm laser (Explorer Laser Spectrum Physics, Mountain View, US), supplies the necessary power to remove the ITO layer. The system (Lasing S.A., Madrid, Spain) consists of a CNC controlled XYZ-stage, with the active focal distance control.

Prior to mounting and filling up the space between the two ITO cells with nematic MDA-98–1600 (Merck KGaA, Germany) LC, both glass wafers are conditioned with polyimide PIA-2304 (Chisso LIXON aligner, Japan) rubbed in an antiparallel manner at 45º to the electrode direction to align homogenously the LC. The mounted LC cells have a measured thickness of 6.4 µm, ensured by using diameter cylindrical silica spacers (HIPRESICA, Japan). The transmission properties of the ITO limits the minimum working wavelength to about 400 nm, while the maximum is determined by the optical retardation variation to about 1500 nm^42^.

### The 2D device

**Figure 3:**
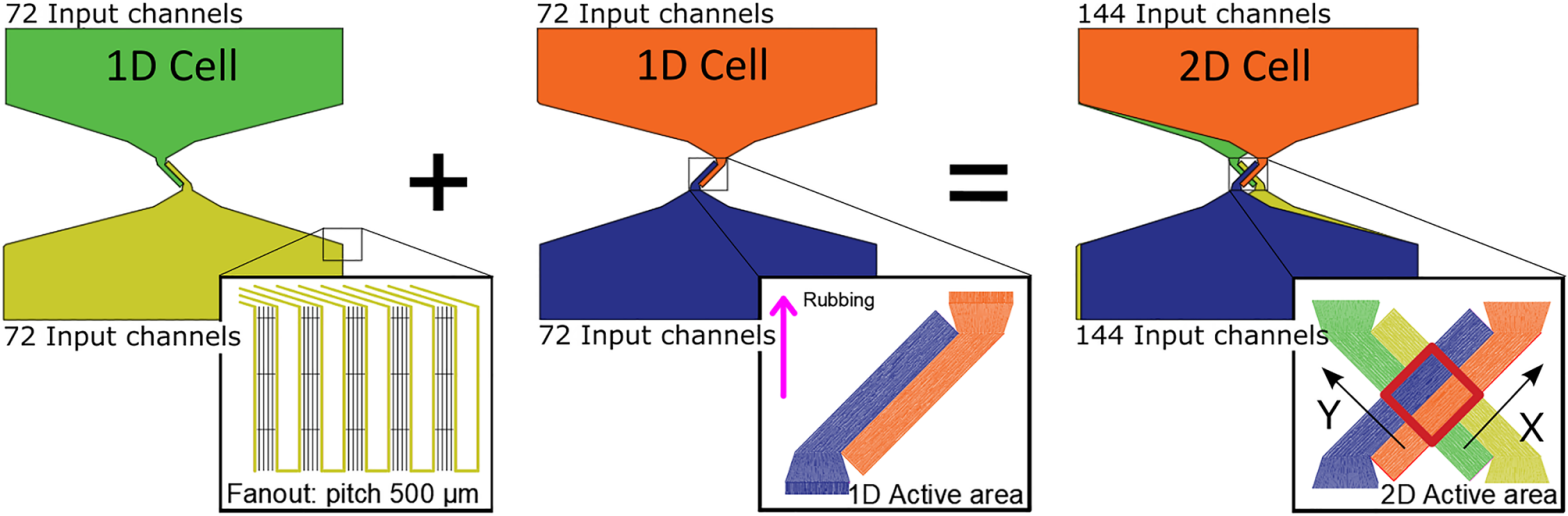
The overlapping of two identical 1D cells, mounted back-to-back, produce the desired 2D steering diffraction pattern. Each 1D cell make used of two 144 input channels by a fanout of 500 µm pitch. The magenta arrow represents the direction of rubbing. A 1 × 1 mm^2^ active area (in red) results from the overlapping. The arbitrarily assigned X and Y directions are indicated.

By cascading two orthogonal 1D LC cells, the 2D effect is achieved (Fig. 3). Hence, one cell deviates the incoming ray in the X direction and the other in the Y direction. Both axes are at 45 degrees to vertical. Each grating structure is made of 144 pixels, with a pitch of 7.5 µm. The width of the pixel is limited by the resolution of the laser ablation. This design leads to an active area of 1.08 × 1.08 mm^2^.

The two 1D cells are identical, in order to simplify the manufacturing process as shown in Fig. 3. The driver used to independently control each electrode in each LC cell has been previously described^53^. This driver has 72 control outputs, so that, it is necessary to daisy-chain two boards to control each of the 144 pixels that make up the 2D device. The AC signal, that is applied to the LC cells, is set to 10 Vpp at 1 kHz. The duty cycle defined by the Pulse Width Modulation (PWM) determines the degree of switching. The PWM cycle is approximately 1 ms.

### Calibration

**Figure 4:**
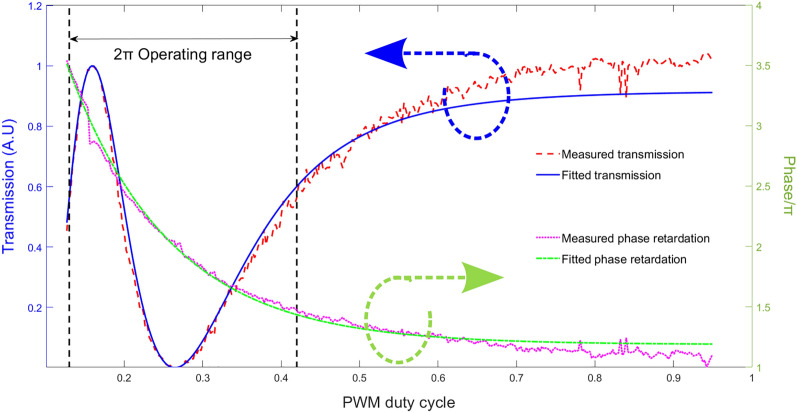
Cell calibration. The normalized transmission intensity measured (red) from the calibration, versus the fitted intensity (blue) as a function of the PWM duty cycle applied. Retardation (phase/π) from measured data (magenta), versus fitted retardation (green), as a function of the PWM duty cycle.

The calibration is performed by placing the 1D device between crossed polarizers with the switching plane at 45º. It establishes the relationship between the induced phase delay in each pixel and the PWM duty cycle (*dc*) as previously described^53^. The phase delay induced by the LC (*δ*) follows a pseudo exponential decreasing function with the applied average voltage of the PWM signal (Eq. 2):

2 $$\delta =A\cdot {e}^{-B\cdot dc}+C$$

in a range [π-x, 3π-x], where x is an offset chosen to ensure the best fit between the fitted curve and the data points while achieving the desired 2π operating range (Fig. 4).

**Figure 5:**
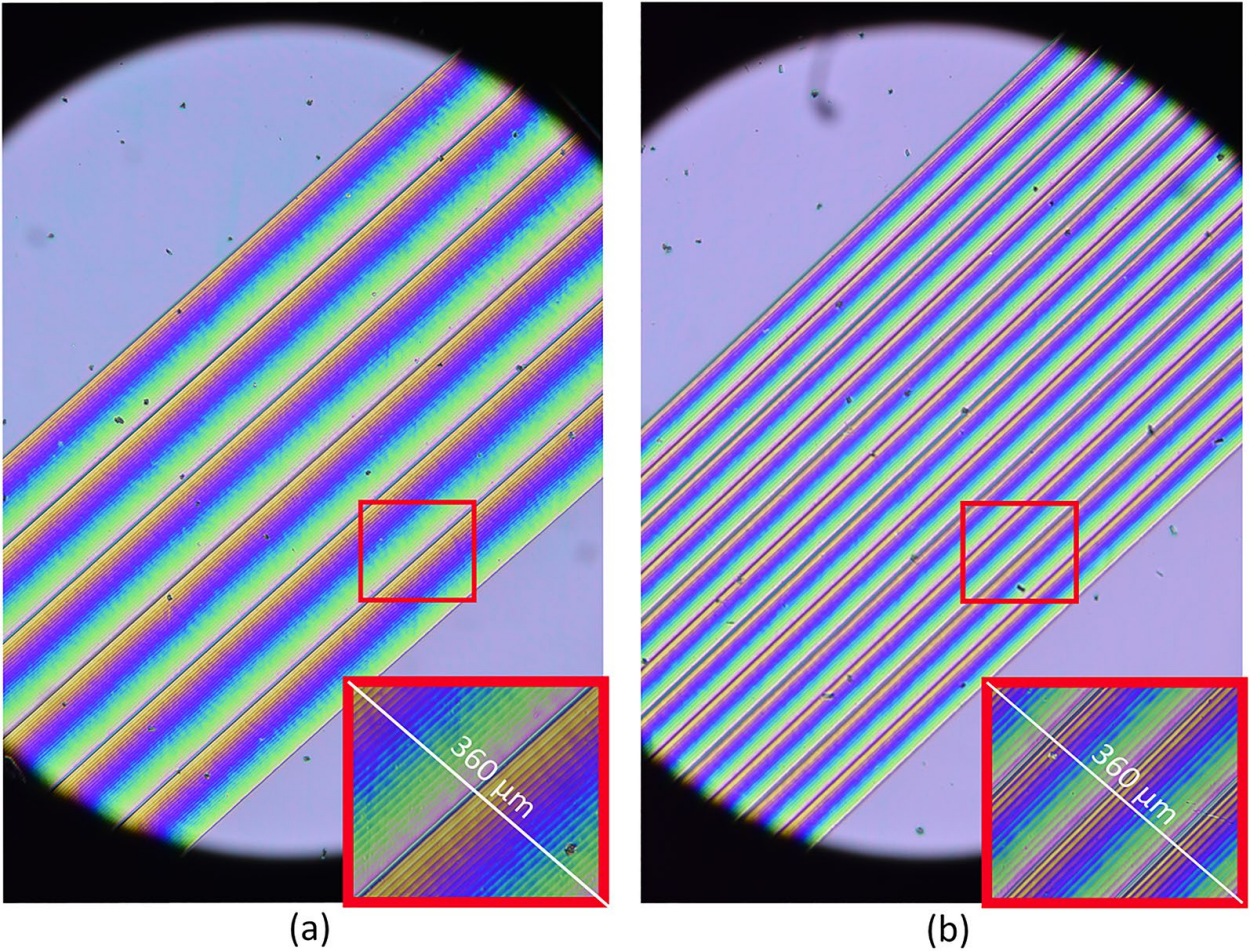
Micrographs of the 1D beam steering active area. (**a**) Micrographs of the active area of sample 1 (1D device), applying an electric field pattern corresponding to a period of 6. Pattern repetition for every 24 pixels, 180 µm. (**b**) Micrographs of the active area of sample 2, applying an electric field corresponding to a period of 12. Pattern repetition for every 12 pixels, 90 µm.

In Fig. 5 micrographs of the active area of two 1D cells (sample 1 and 2, respectively) between cross polarizers are shown. The period applied to one cell (Fig. 5a), correspond with half the period of the other (Fig. 5b). One may appreciate the colored symmetry of the periods (2:4) in the phase profile.

## Results and discussions

Three aspects of the system are studied: the angular resolution, the diffraction efficiency and the spot shape variation. The experimental setup, similar to that previously presented^42^, is provided in the supplementary information.

**Figure 6:**
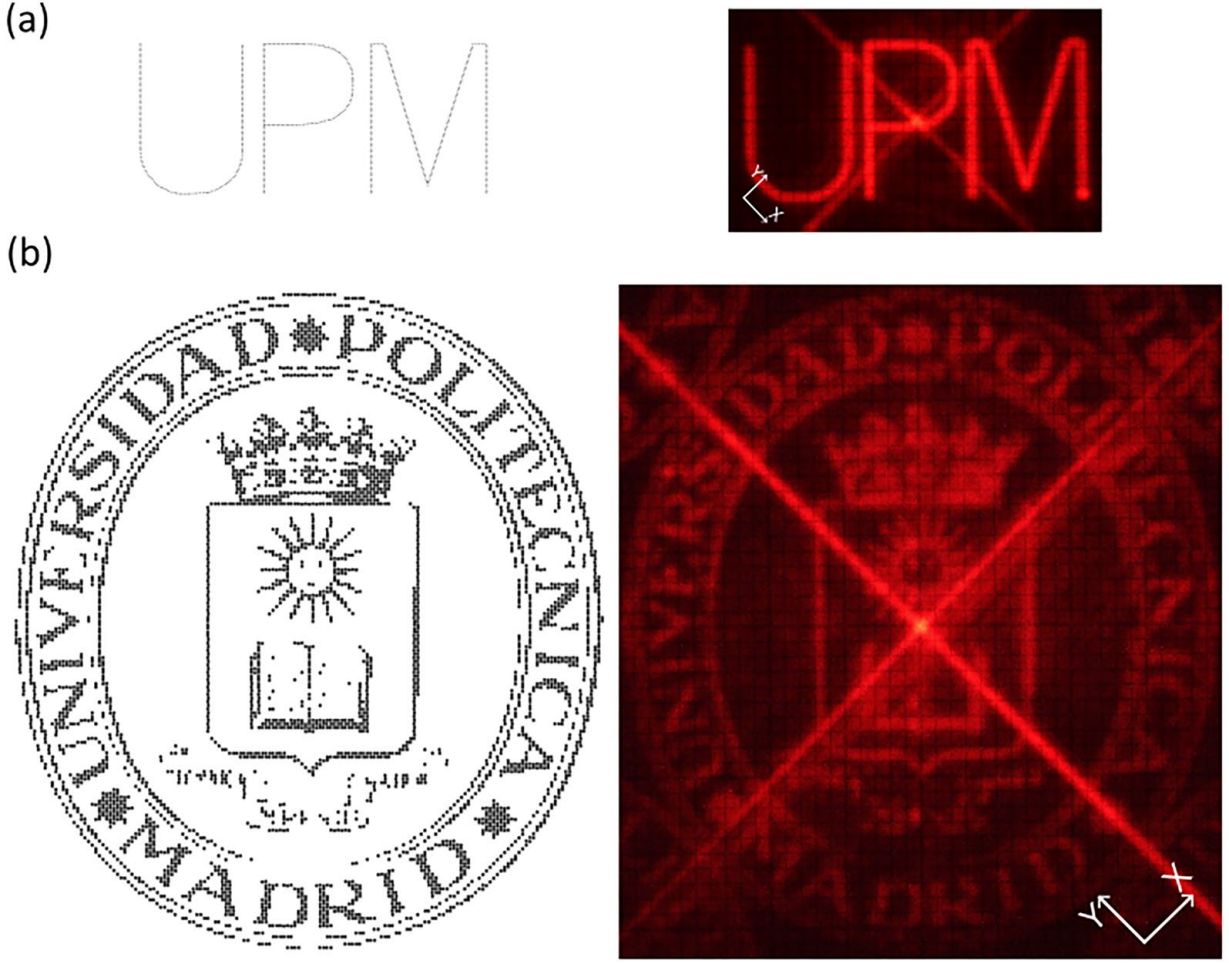
Performance of the 2D diffractive beam steering. (**a**) “UPM” logo. Comparation between the input image (548 points) and the 2D steered map over a screen. (**b**) “UPM shield”. Comparation between the input bitmap (4718 points) and the 2D steered map over a screen. The distance between the screen and the steerer was 50 cm.

Figure 6 shows the performance of the 2D beam steering device. The deflected beam is projected onto a millimeter screen at a distance of *d* = 50 cm from the BS. The camera (NIKON D500) is focused on the screen with the shutter open. The beam steerer creates the image point by point. The image capture lasted 150 and 1353 s, respectively. Hence, the resulting photos, Fig. 6a,b, are the combination of sequential points and their transitions, created with the 2D steerer system.

Every spot diffracted is the result of the diffraction pattern of two 1D LC cells. The bright diagonal cross and the central spot, that appears in Fig. 6a,b, correspond to the non-diffracted light in the two cells. The bright central spot, in which light passes the device without being diffracted, corresponds to the integration of the 0th order in both directions. The cross corresponds the 0th order diffraction in only one of the two directions.

In Fig. 6a subsequent points are separated 0.6 periods since this corresponds approximately to the 1 mm spot size at a distance of 50 cm between the steerer and the screen. The position of each point is defined to the fourth decimal of a period, *p*. For the given *p*, the phase profile is calculated, and the desired phase delay for each of the electrodes is derived. The resulting PWM of each electrode is determined from the calibration curves. Figure 6b is simply the 1:1 imaging of a 144 × 144 bitmap, where each pixel is situated at the integer period diffraction points. The images show how the system is capable of performing a continuous deviation by using integer and non-integer periods.

The resolution of the developed device is limited by the 12-bits Digital to Analog Converter (DAC) of the driver. The full range of the DAC corresponds to a fully relaxed (maximum retardation) to an almost saturated cell (minimum retardation), in the case of the 6.4 µm thick cells this corresponds to a 6π retardation difference, as may be appreciated in Fig. 4. But for the grating to work, only a phase retardation of 2π is needed, and thus we only use a fraction of the full DAC range.

In practice, the 1D cell is calibrated over a range of approximately 1200 PWM values. So that, the theoretical minimum phase step that the driver can provide is λ/1200. According to that, applying a different phase step to each pixel, the minimum interval for a continuous retardation is $\Gamma =144\cdot \lambda /1200$. That corresponds to a deviation angle of approximate $0.12\cdot \lambda /D$ (being D the diameter of a circular aperture), which is significantly less than the aperture diffraction limit of $1.22\cdot \lambda /D$. The presented steerer is, by grouping electrodes, capable of resolutions of $(\lambda /1200) /D$. Simply replicating the active area by interconnecting multiples of electrodes separated 1.08 mm, would mean that only integer *p* values could be employed, like in other works^43^, and thus the resolution will be limited to $\lambda /D$. To what extent a steering resolution finer than the device aperture diffraction limit, will depend on the final application. In the setup, the input spot diameter is limited to 1 mm.

The relaxation time of the manufactured device is in the order of 10 s of milliseconds, as can be expected from such a relatively thick LC cell made of standard material. Neither the driver nor the cells have been designed with the response time in mind. However, recent studies by others, employing vertically aligned nematic LCs have reported response times of 2 ms for similar devices^54^.

**Figure 7:**
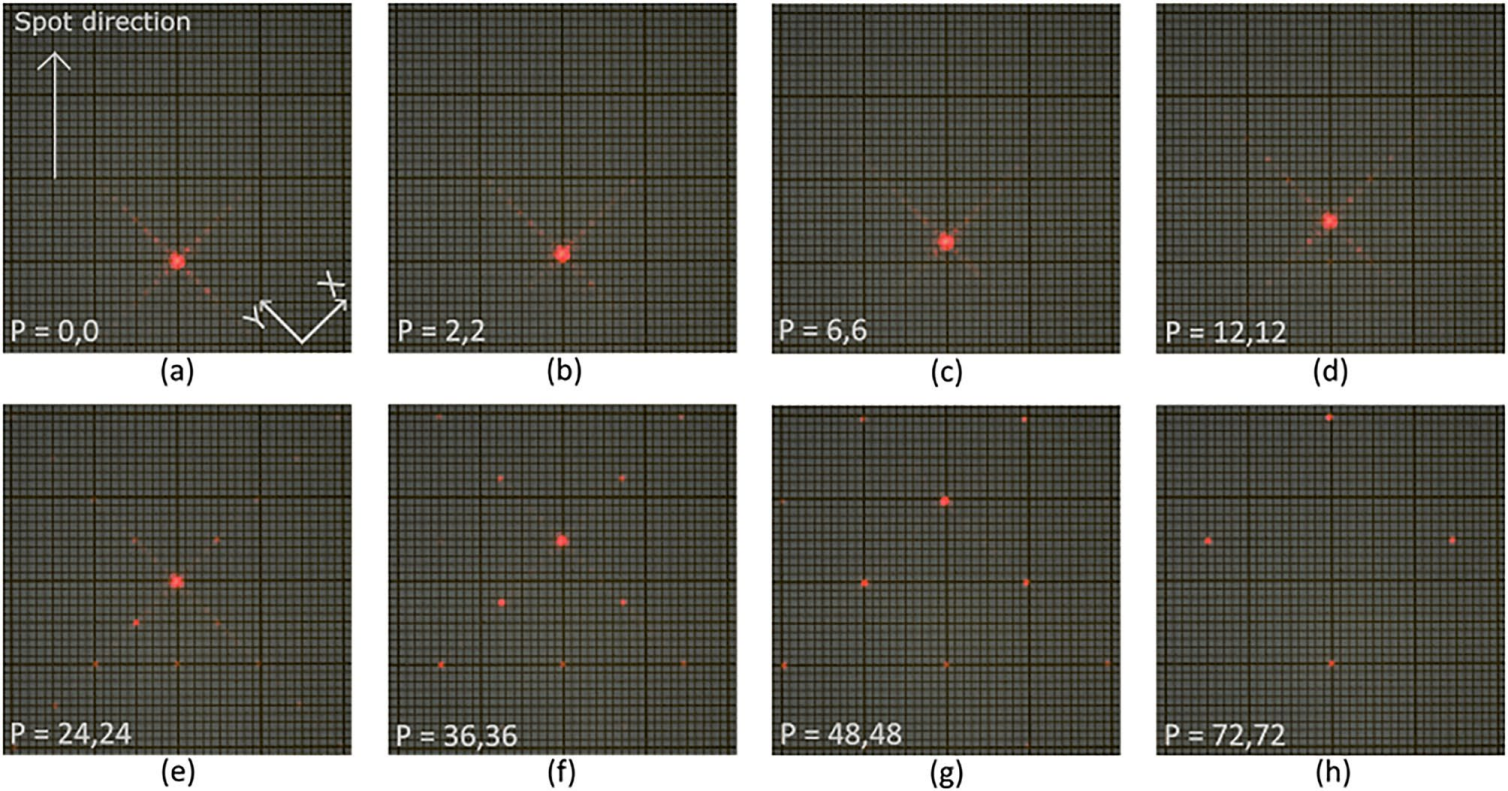
View of the diffraction patterns of the 2D BS, applying representative phase grating periods symmetrically in two 1D cells. The millimeter screen is situated 50 cm from the device. (**a**)Periods = 0. (**b**) Periods = 2. (**c**) Periods = 6. (**d**) Periods = 12. (**e**) Periods = 24. (**f**) Periods = 36. (**g**) Periods = 48. (**h**) Periods = 72.

In Fig. 7, the performance of the device is presented for a representative number of periods. In this case, both 1D cells make use of the same configuration. A maximum angle of 3.42 degrees, along the vertical direction (combining the two gratings), is achieved in the binary configuration of the grating, corresponding with periods equal to 72. Notice that a weak, or absent, central 0º order peak, except for period = 0, indicates that the desired phase pattern has been achieved^54^.

One can appreciate in Fig. 7, that the closer the grating is to the binary configuration (*p* = 72, identical to *p* = -72, Fig. 7h) less is the intensity of the 1^st^ order. This is in accordance with diffraction theory, which predicts a 40% diffraction efficiency for a perfect 1D binary diffraction grating; thus, in the case of the 2D grating, the efficiency becomes 16%. Depending on a final application this diffractive limitation may be acceptable or not.

The spot intensity shape has been analyzed for the 2D system. To do so, the beam was shone directly into the camera sensor at a distance of 7 cm from the device.

**Figure 8:**
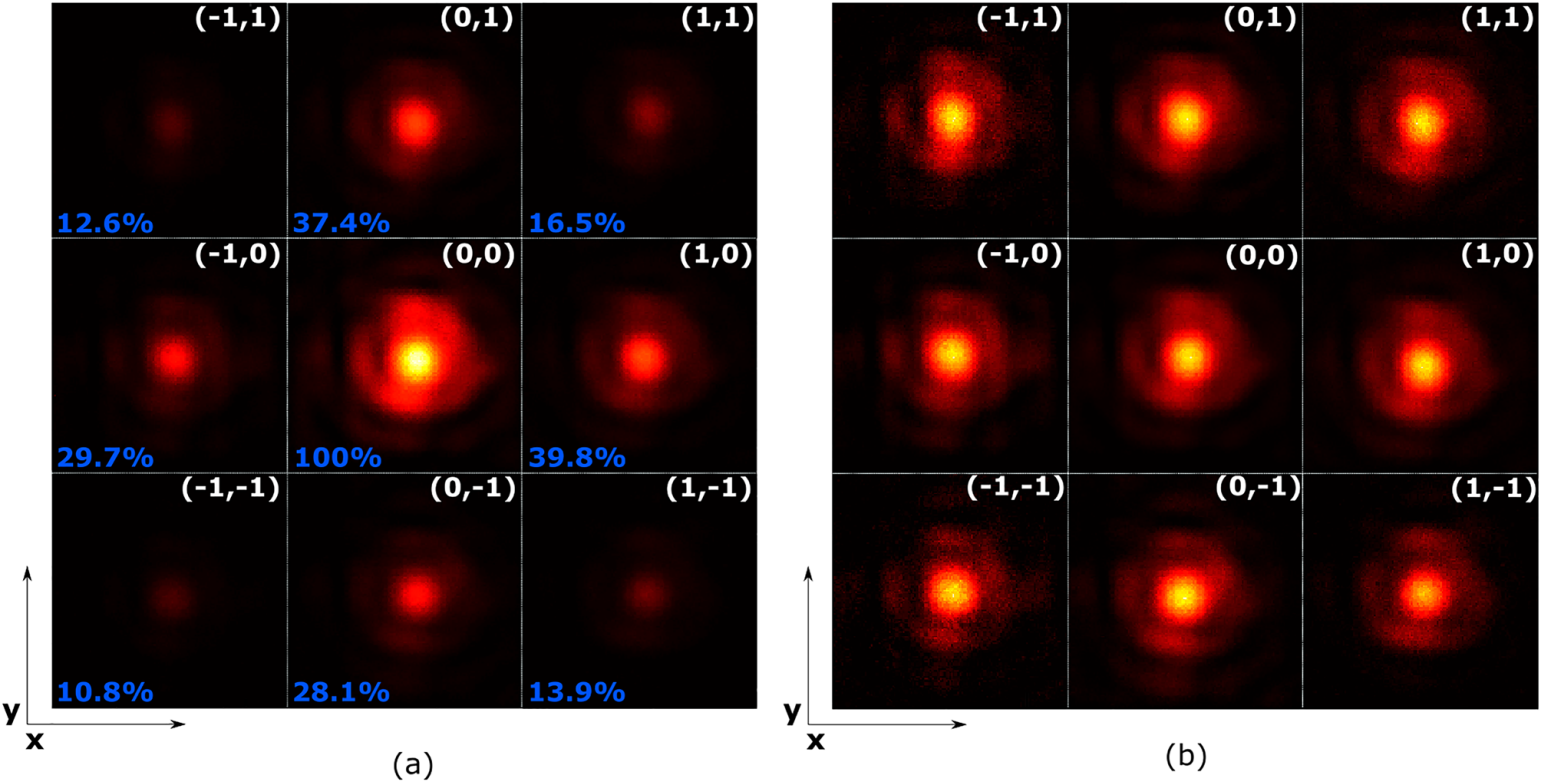
Images of the deviated spot for the extreme periods 0 and ± 72. The symmetry of the binary pattern means that in the first approximation two opposite spots (denoted ± 1 should be of the same intensity). In the four corners both 1D cells exhibit the binary diffraction pattern, in the four vertices only one. (**a**) Comparison of the spot intensity for the different binary combinations. The images have been normalized to the intensity in p = (0,0). The diffraction intensity is indicated in blue. (**b**) Comparison of the spot shape for the different binary combinations, each sub-image has been normalized individually. Notice that the images are from separate distant parts of the detector and have been rotated 45º with respect to Fig. 7, hence the rubbing direction is along the vector joining the third (1,1) and sixth (− 1, − 1) points in these images.

In Fig. 8 eight representative points were captured. Deviation corresponds to the binary pattern in either or both of the 1D devices. Hence, Fig. 8a,b are divided into nine parts. In the (0,0) image, period = 0, is the origin of the unit XY axis, and shows the quality of the non-steered light beam. The other pictures correspond to the binary configurations (*p* = 72), in one 1D cell or in both. All images have been processed and resized in the same proportion (as is detailed in the supplementary information).

In the setup, the input spot diameter is limited by a 5-level diaphragm, i.e. the input spot is not perfectly circular, as is reflected in all images.

Figure 8a shows reduced their efficiency in the corner images, in accordance with the diffraction theory. The nine images are pictured with the same intensity scale. In the case of pure X or Y deviation angles, the efficiency is reduced less than in the vertical/horizontal diagonals since only one cell is generating the diffraction deviation while the other is in the OFF state with no diffractive pattern.

From the images one may appreciate an asymmetry in the intensity of the spots between counterpart points. Points in the first quadrant have more intensity that points in the third quadrant. These differences are tangible in Fig. 6. This is attributed to the pretilt applied to the LC cells, result of the rubbing process, that is along the diagonal in this figure. Asymmetric response caused by rubbing has been described recently by others^55^. It is possible that the effect may be alleviated by employing parallel rather than antiparallel rubbing, which will be the subject of a future study.

The efficiency of each image (Fig. 8a) has been calculated, normalized with the central spot (*p* = 0). The diffraction efficiency does not quite correspond to the theoretical 40% and 16% diffraction efficiency in 1D and 2D binary gratings respectively, which is attributed to the abovementioned asymmetric switching and the fringe effects caused by the interpixel space and the elastic constants of the LC.

Figure 8b shows the same points, but intensities has been normalized individually. Here one can appreciate that the spot shape is maintained even as the deviation angle is increased.

## Conclusions

A high resolution 2D beam steerer device, based on a LC blazed grating with individual control of each pixel has been manufactured and demonstrated by projection onto a screen, and by direct projection into a camera image sensor. The device is characterized by not having any electronics in nor below the active area, which makes it suitable for applications in harsh conditions such as intersatellite communications.

The device, manufactured by cascading two identical back-to-back 1D beam steerers, has shown an, to our knowledge, unprecedented diffraction resolution, and a minimal spot shape distortion.

The individual control of all pixels in the device leads to a diffraction resolution, not previously presented in passively addressed LC devices, which combined with the 3.42º diagonal steering angle (at the specific wavelength) leads to a very versatile optical component.

## Supplementary Information

Supplementary Information.

## Data Availability

All data generated or analyzed during this study are included in this published article (and its Supplementary Information files).
