# Supplementary material for: High resolution 2D beam steerer made from cascaded 1D liquid crystal phase gratings
Source: Sci Rep. 2022 Mar 24;12:5145. doi: 10.1038/s41598-022-09201-0 (PMC8948363; doi:10.1038/s41598-022-09201-0)
Supplement: Supplementary file 1 — Supplementary Information. [file 41598_2022_9201_MOESM1_ESM.zip › Supplementary information/supplementary information_videos/Legends for the supplementary videos.pdf]

Legends for the supplementary videos

1. Circular trajectory.mov
   This file illustrates the 2D beam steerer performance. Every spot represents one point of a circular trajectory (36 points in total).

2. Circular trajectory-shutter time 20s.jpg
   This file corresponds with a picture of the 2D beam steerer performance. The beam steerer is performing the same circular trajectory as in the video (Circular trajectory.mov). In this case, the shutter time of the camera is 20s, so that, every point is integrated in the image.

3. Linear trajectory.mov

   This file illustrates the beam steering performance, changing periods from -72 and 72 in every 1D cell.
