# Supplementary material for: High resolution 2D beam steerer made from cascaded 1D liquid crystal phase gratings
Source: Sci Rep. 2022 Mar 24;12:5145. doi: 10.1038/s41598-022-09201-0 (PMC8948363; doi:10.1038/s41598-022-09201-0)
Supplement: Supplementary file 1 — Supplementary Information. [file 41598_2022_9201_MOESM1_ESM.zip › Supplementary information/supplementary_info.docx]

High resolution 2D beam steerer made from cascaded 1D liquid crystal phase gratings.

Mario García de Blas*, Javier Pereiro García, Sergio Vera Andreu, Xabier Quintana Arregui, Manuel Caño-García and Morten Andreas Geday*

CEMDATIC, ETSI Telecomunicación, Universidad Politécnica de Madrid, Av. Complutense 30, 28040 Madrid, Spain.

# Supplementary information

### Diffraction Efficiency figure composition

The Figure S1 shows the intensity distribution of a laser spot when deflected due to the 2D beam steering to the maximum allowed angles and the diffraction efficiency. Thus, the difference between the 0^th^ order intensity and the intensity in the outer points could be compared.


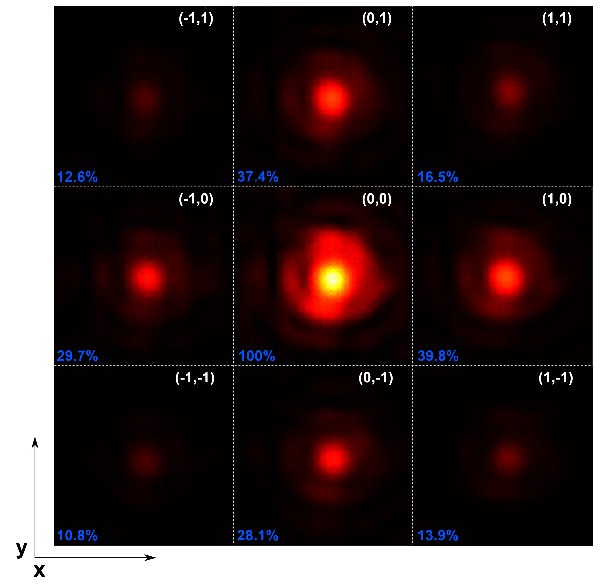


Figure S1: Images of the deviated spot for the extreme periods 0 and ±72. The symmetry of the binary pattern means that in the first approximation two opposite spots (denoted ±1 should be of the same intensity). In the four corners both 1D cells exhibit the binary diffraction pattern, in the four vertices only one. Comparison of the spot intensity for the different binary combinations. The images have been normalized to the intensity in p = (0,0). The diffraction intensity is indicated in blue.

This Figure S2 is a composition of 9 pictures taken by a Nikon D500 camera ^1^ which has been made using both MATLAB ^2^ and Inkscape^3^. In this way, to obtain the mentioned composition, the next steps were carried out:

1. Importing all the NEF pictures into a uint16 numerical array.
2. The pixels corresponding to the camera Gx channel are obtained and used to build a squared image.
3. A mask matching the squared picture size is created. This mask is divided in 9 equal cells.
4. Each image is multiplied by the mask matrix where there is only one active cell corresponding to the region of interest. The result is stored as an array.
5. The step four is repeated until all the images are added into the resulting array.
6. Once the composed image containing the region of interest of each one of the 9 pictures is formed, the vector graphics editor is used to apply zoom to the spot region.


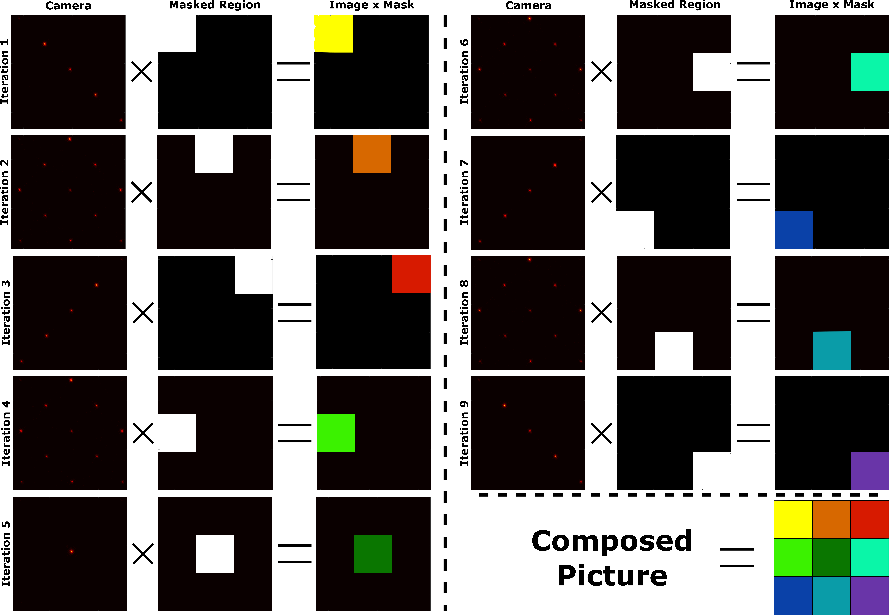


Figure S2: Composition process scheme where the nine iterations are displayed.

Moreover, to calculate the diffraction efficiency of each region in Figure S1, the next process was carried out. First, the camera pixel minimum value of each region of interest (composed by an image multiplied by its corresponding mask) is subtracted. This step is required to compute the correct efficiency values, because the camera produces a pixel value of around 400 even if they are in total darkness. Then, the intensity is calculated by adding the pixel values of the whole region of interest divided by the number of pixels. Lastly, the diffraction efficiency of each region is obtained by dividing its intensity by the maximum intensity region, corresponding to the central region where light is not diffracted.

Regarding the Figure S3, the composition process is essentially the same. However, the resulting region of interest intensity is normalized to each region maximum intensity, in opposition to the previous case were all of them were normalized to the 0^th^ order intensity. In addition, after scaling each region it is required to remove the background. Once both steps are performed, all the regions are composed in the same way as the Figure S1. Thus, this intensity scaling enables the spot shape comparison between the different steering angles compared to the 0^th^ order.


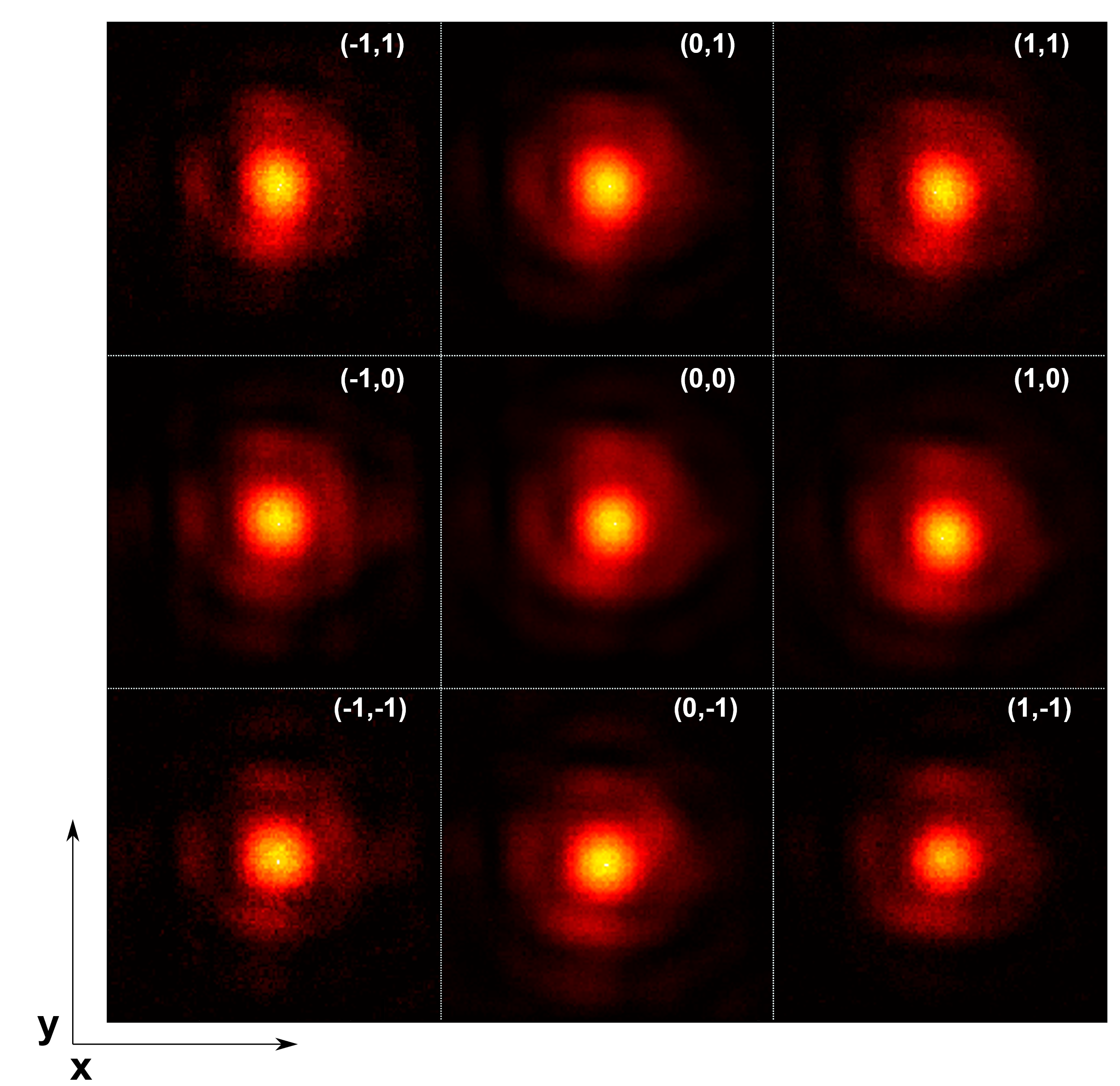


Figure S3: Images of the deviated spot for the extreme periods 0 and ±72. The symmetry of the binary pattern means that in the first approximation two opposite spots (denoted ±1 should be of the same intensity). In the four corners both 1D cells exhibit the binary diffraction pattern, in the four vertices only one. (b) Comparison of the spot shape for the different binary combinations, each sub-image has been normalized individually.

### Experimental setup

### The setup is presented in Figure S4 and Figure S5. A He-Ne laser with a wavelength of 632.8 nm is used as input ray beam, as is collimated by a collimator adjacent to the laser system. To reduce the diameter of the spot to the desired one (1mm, according to the active area of the 2D beam steerer) a diaphragm is used. Then, the incoming beam pass through a polarizer in order to ensure the proper axis of polarization (90º, making it coincident with the rubbing direction). Two 1D cells are joint together and placed in a holder, resulting in the final 2D device.


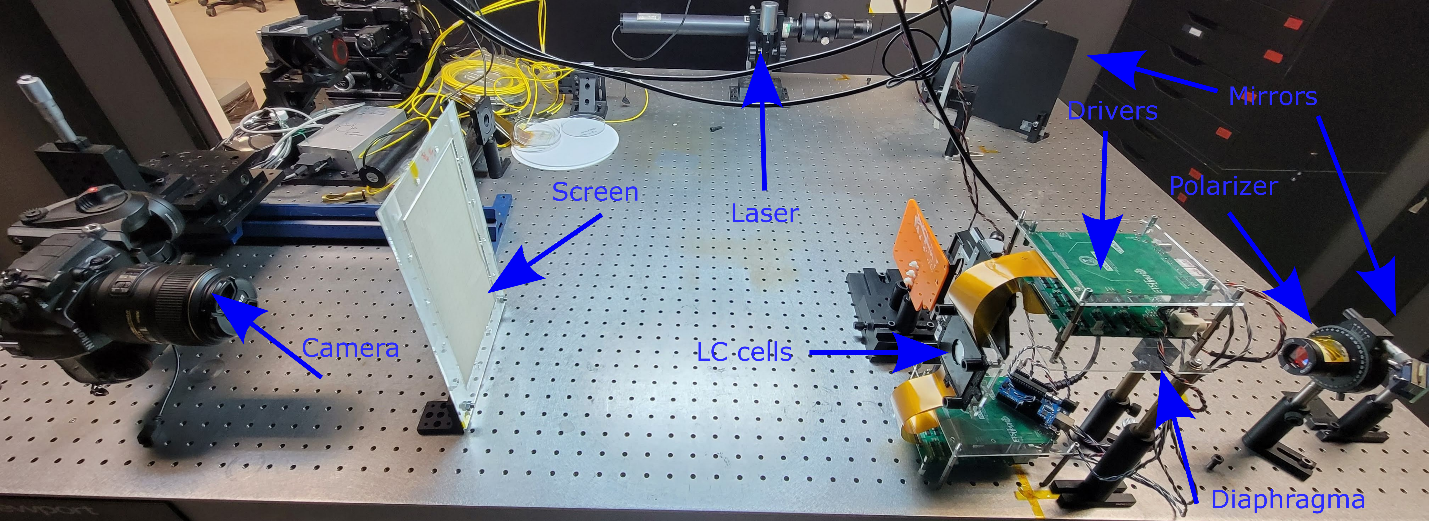


Figure S4: Overview of the setup.


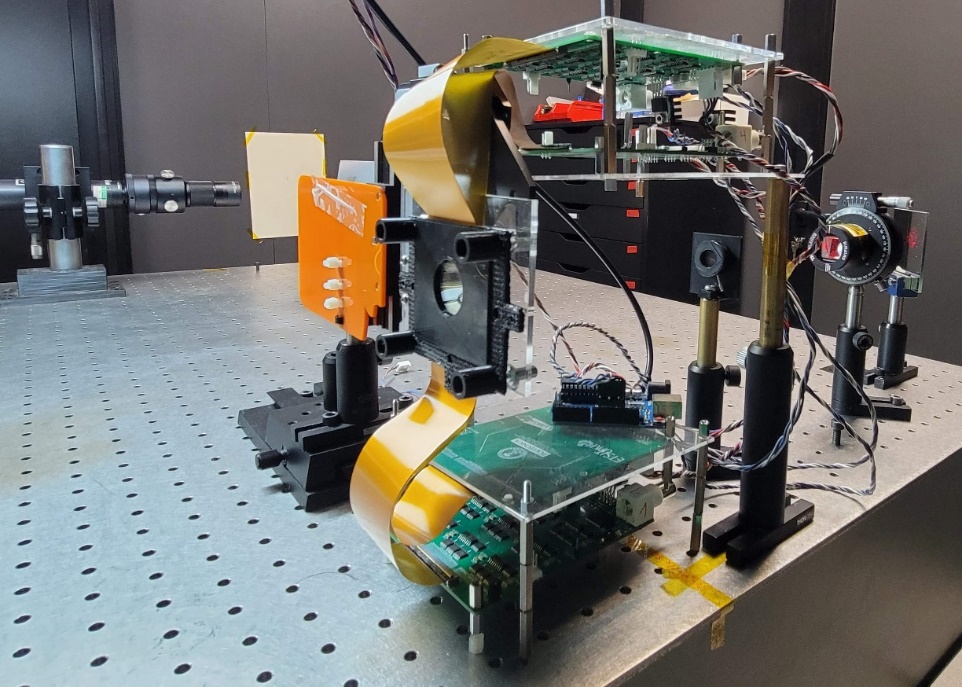


Figure S5: Setup with the LC cell (In their holder), the driver, the diaphragm, the polarizer, and the laser input.

### Direct Laser Writing setup characteristics

The employed setup is based on optical XYZ stages and provides positional precision better than 0.5 µm.

The ablation has been done using back scribing, focusing the laser beam at the ITO. The laser is a pulsed laser working at 50 kHz. This ensures an experimentally determined minimum linewidth of ablation of less than 2 µm, when approximately 20 % of the total laser power is employed. However, to ensure no shortcuts between electrodes, and thus higher yield, the ablation has been done at twice this power, leading to linewidths of approximately 3.0 µm.

The ablation was done a translation speed of up to 5 mm/s, which means that the active area, characterized by 145 lines of 7 mm was written in approximately 5 minutes. The fanout, that was written with 10 mm/s and 100 pct power took a lot longer, since hatching of the interelectrode area was done to minimize risk of shortcuts outside the active area.

### References

1. Nikon D500 | Cámara DSLR de formato DX | SOY RENDIMIENTO CONCENTRADO. https://www.nikon.es/es_ES/product/digital-cameras/slr/professional/d500.

2. MATLAB - El lenguaje del cálculo técnico. https://es.mathworks.com/products/matlab.html.

3. Developers, I. W. Draw Freely | Inkscape. https://inkscape.org/.
