# Supplementary figures and images for: High resolution 2D beam steerer made from cascaded 1D liquid crystal phase gratings
Source: Sci Rep. 2022 Mar 24;12:5145. doi: 10.1038/s41598-022-09201-0 (PMC8948363; doi:10.1038/s41598-022-09201-0)

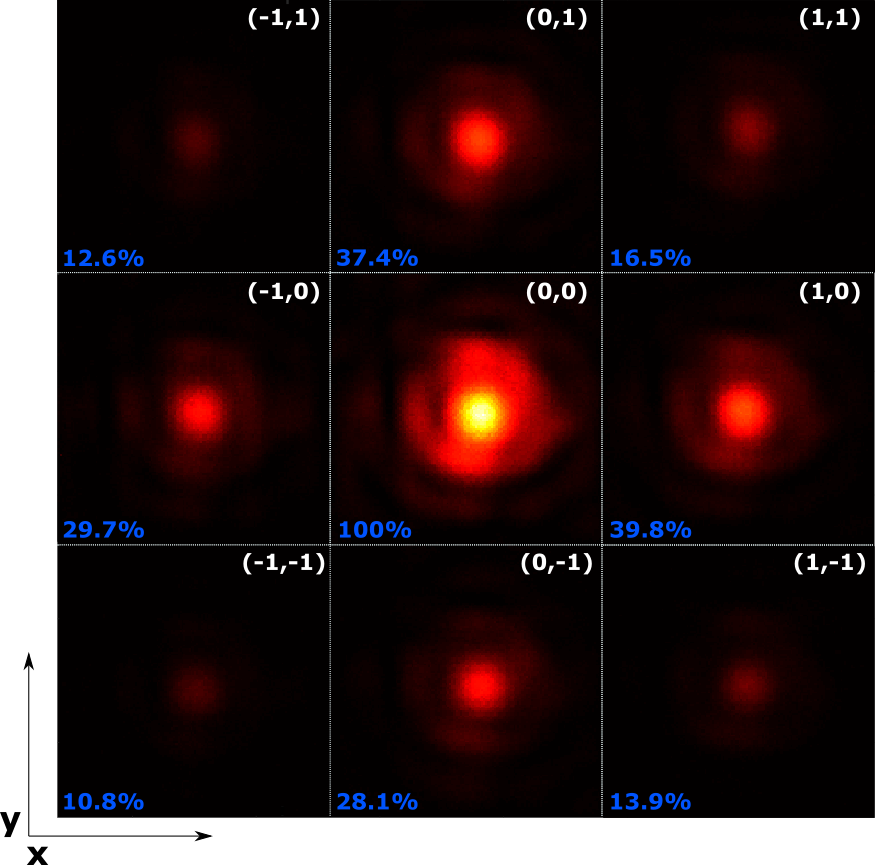

Supplement: Supplementary file 1 — Supplementary Information. [file 41598_2022_9201_MOESM1_ESM.zip › Supplementary information/Figures supplementary info_document/Figure S1_supplementary.png]

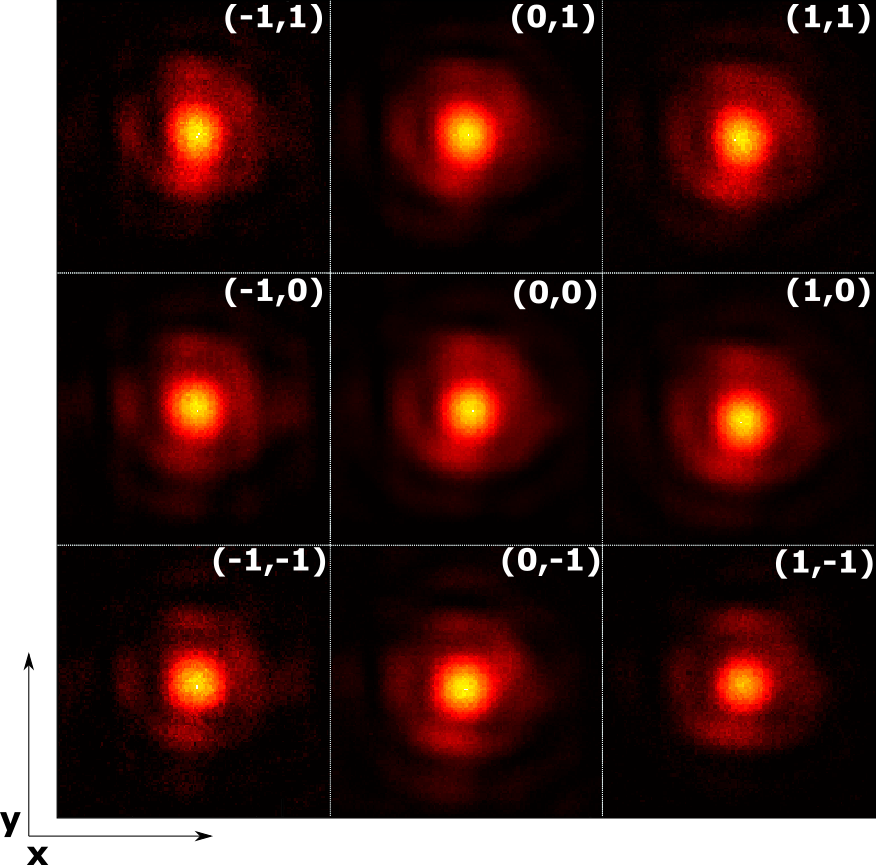

Supplement: Supplementary file 1 — Supplementary Information. [file 41598_2022_9201_MOESM1_ESM.zip › Supplementary information/Figures supplementary info_document/Figure S3_ supplementary.png]

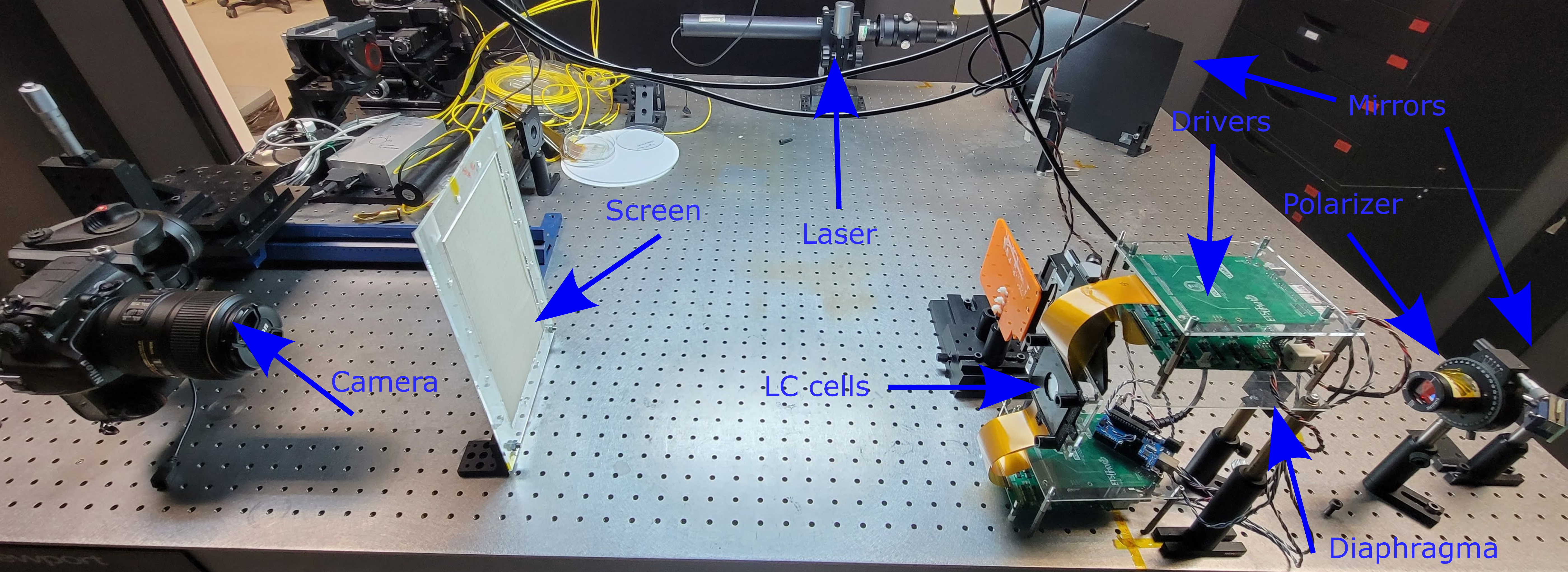

Supplement: Supplementary file 1 — Supplementary Information. [file 41598_2022_9201_MOESM1_ESM.zip › Supplementary information/Figures supplementary info_document/Figure S4_supplementary.png]

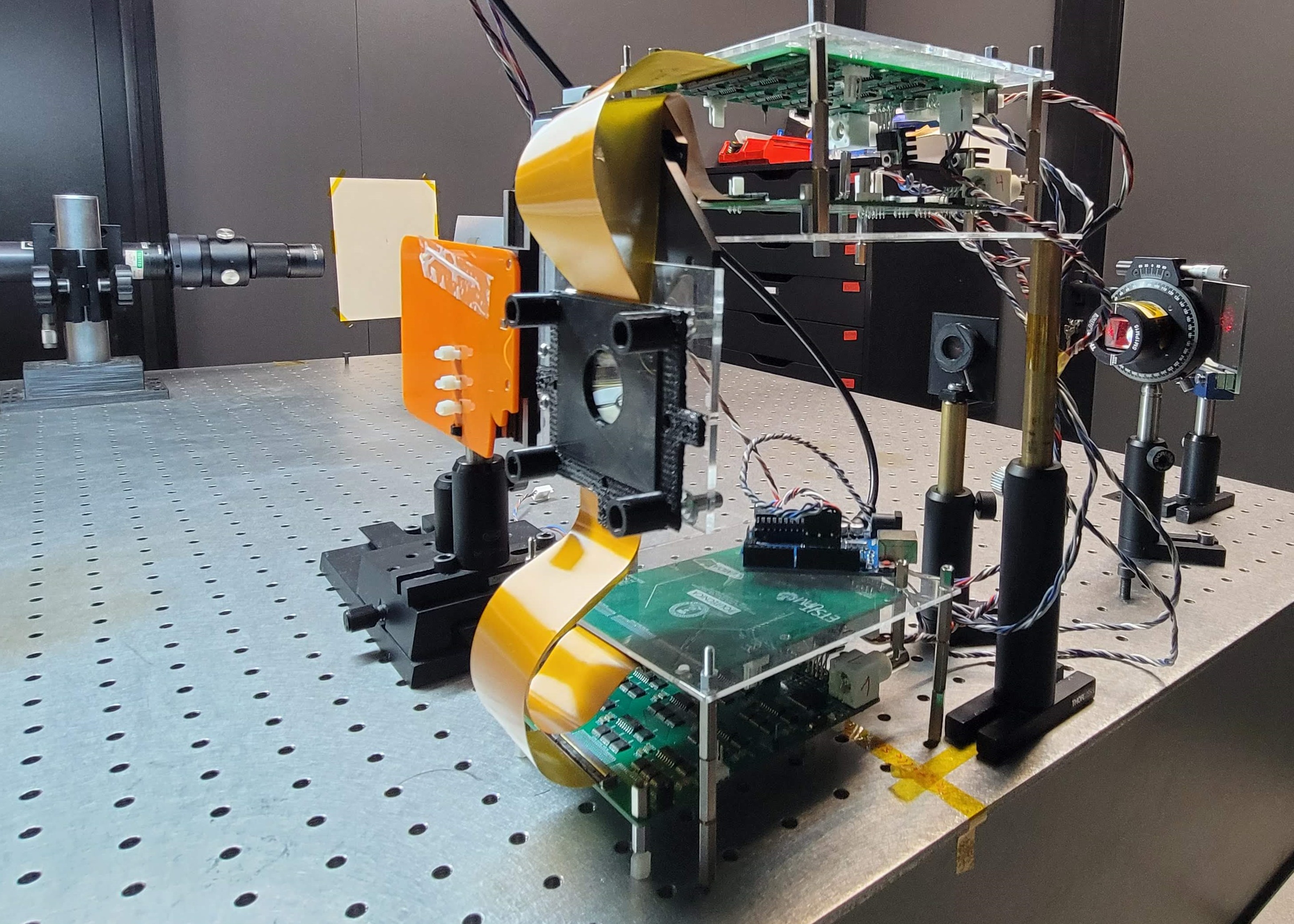

Supplement: Supplementary file 1 — Supplementary Information. [file 41598_2022_9201_MOESM1_ESM.zip › Supplementary information/Figures supplementary info_document/Figure S5_supplementary.jpg]

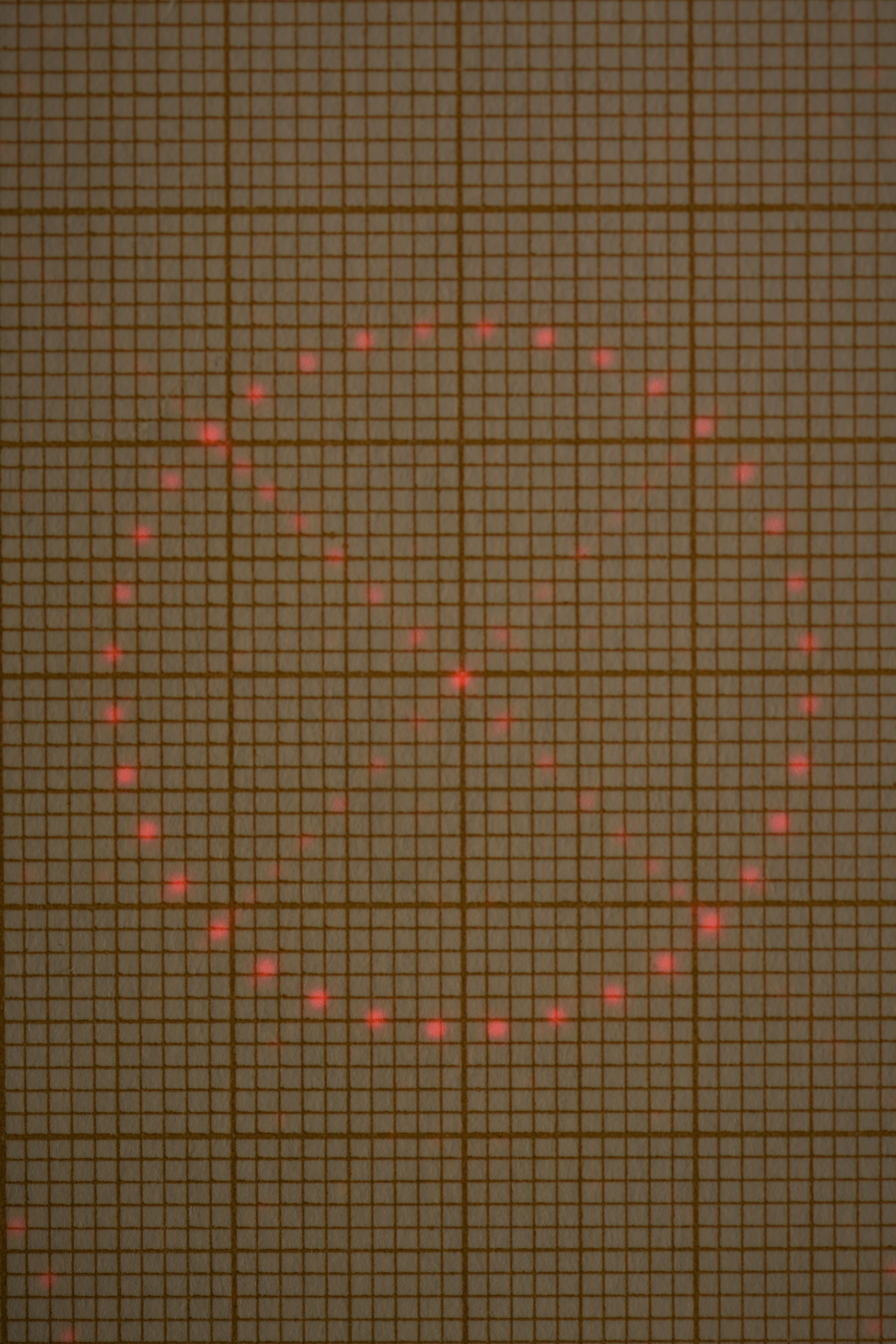

Supplement: Supplementary file 1 — Supplementary Information. [file 41598_2022_9201_MOESM1_ESM.zip › Supplementary information/supplementary information_videos/Circular trajectory - shutter time 20s.jpg]
